# Drug-resistant tuberculosis in Central Mozambique: the role of a rapid genotypic susceptibility testing

**DOI:** 10.1186/s12879-016-1766-x

**Published:** 2016-08-17

**Authors:** Evangelina Inácio Namburete, Inês Tivane, Miguelhete Lisboa, Margarida Passeri, Renata Pocente, Josefo Joao Ferro, Lee H. Harrison, Valdes Roberto Bollela

**Affiliations:** 1 School of Medicine of Ribeirão Preto, University of São Paulo, Ribeirão Preto, Brazil; 2 Laboratório de Referencia de tuberculose, Hospital Central da Beira, Moçambique, Beira, Mozambique; 3 Laboratório de Microbactérias do HCFMRP, USP, Ribeirão Preto, Brasil; 4 Faculdade de Ciências de Saúde da Universidade Católica de Moçambique, Beira, Mozambique; 5 Infectious Diseases Epidemiology Research Unit, University of Pittsburgh, Pittsburgh, USA; 6 Internal Medicine Department, Hospital das Clínicas School of Medicine of Ribeirão Preto, University of São Paulo, Avenida Bandeirantes 3900, Monte Alegre, 14049-900 Ribeirão Preto, São Paulo Brazil

**Keywords:** Tuberculosis, Molecular diagnosis, Mozambique

## Abstract

**Background:**

Genotypic molecular testing may be very helpful for tuberculosis (TB) drug-resistance surveillance and for treatment guidance in low resource settings.

**Methods:**

Descriptive analysis of *M. tuberculosis* isolates from Beira Central Hospital, Mozambique, during 2014–2015. Genotype MTBDRplus and MTBDRsl were used and patient medical records reviewed.

To explore genotypic susceptibility profile of *Mycobacterium tuberculosis,* to first and second line drugs (SLD) in Beira Mozambique.

**Results:**

Of 155 isolates, 16.1 % (25) were multidrug resistant (MDR), 8.4 % (13) isoniazid-monoresistant and 1.3 % (2) rifampicin-monoresistant. Among MDR-TB, 22.2 % showed primary and 77.8 % represented acquired resistance. The majority of patients with drug resistance had a history of previous TB treatment. Among 125 isolates tested for ethambutol and SLD, 7.2 % (9) were resistant to ethambutol, 4.8 % (6) to fluoroquinolones and 0.8 % (1) to ethambutol and fluoroquinolones. Resistance to injectable SLD was not detected.

**Conclusions:**

As far as we know this is the first report of a genotypic testing used to provide information about SLD resistance in Mozambique, where phenotypic susceptibility testing is usually unavailable. Extensively drug resistant TB was not detected in this isolates from Beira Mozambique.

## Background

Drug resistant tuberculosis (DR-TB) is a major threat worldwide. It is estimated that 3.5 % of new cases and 20.5 % of previously treated cases have multidrug resistant TB (MDR-TB) and almost 9 % of MDR-TB patients are carrying extensively drug resistant TB (XDR-TB) worldwide [1–4].

Sub-Saharan Africa is a region with the highest TB burden, where the numbers of DR-TB and HIV coinfection are increasing continuously [1, 5]. In Mozambique, drug resistance is a great challenge for the National Program of TB Control (NPTBC). From 2010 to 2014, NPTBC reported 482 DR-TB cases but there is scarce information about the occurrence of XDR-TB in the country. Data from a national drug resistance survey indicated that 3.5 % of DR-TB in the country is primary resistance while 11.6 % is secondarily acquired [6, 7]. These numbers may be an underestimate because of the lack of laboratory facilities for phenotypic drug susceptibility test (DST) in the country, especially for second line TB drugs (SLDs).

The diagnosis of DR-TB requires susceptibility testing to anti-TB drugs, either by phenotypic DST or rapid molecular tests such as Line Probe Assay (LPA). Genotype MTBDRplus is an LPA recommended by WHO for DR-TB surveillance and diagnostic worldwide [8]. Genotype MTBDRplus evaluates resistance to both isoniazid (H) and rifampicin (R), which is the definition of MDR-TB. The test also classifies resistance to H according to the mutation detected: high level resistance (mutation in *katG gene)* and low level resistance (mutation in *inhA* gene regulatory region) [9]. In Mozambique this test is available only at the National Laboratory of TB in the capital city, Maputo.

Genotype MTBDRsl is the only commercial rapid molecular test available for detection of resistance to the SLDs. The WHO Expert Group recommended that the Genotype MTBDR*sl* assay cannot be used as a replacement test for conventional phenotypic DST, but it may be used as a rule-in test for XDR-TB while waiting for confirmatory results [10–13]. This test has low sensitivity especially for detection of resistance to injectable SLD. When used, Genotype MTBDR*sl* can reduce dramatically the delay to the initiation of adequate therapy, as it detects 2 in 3 or even 3 in 4 of XDR-TB cases [10].

Treatment of MDR-TB in Mozambique is standardized with 6 classes of anti-TB drugs (kanamycin/capreomycin, levofloxacin/ofloxacin, ethionamide/prothionamide, cycloserine, ethambutol and pyrazinamide) [14].

Timely information provided by LPAs regarding to the first- and second-line TB drugs would be helpful for individual decision-making and for review and improvement of the DR-TB management protocols in Mozambique. The aim of this study was to evaluate the role of the genotypic susceptibility testing to the first- and second-line drugs in surveillance and treatment of MDR- and XDR-TB.

## Methods

### Study population

This is a descriptive study, where patients diagnosed with TB, confirmed with liquid medium (MGIT) culture at the Tuberculosis Reference Laboratory in Beira Central Hospital, Beira, Mozambique, from January 2014 to March 2015 were analyzed. Only one isolate of *Mycobacterium tuberculosis* per patient was tested. If a patient had more than one isolate, the first one was included in the study.

### Data collection

Specimens were collect during routine diagnostic investigation of TB buy the clinical team that was taking care of the patient, according to the routine of the laboratory: direct exam after auramine O staining was done, and then the sample was incubated in liquid medium culture in an MGIT 960 automated system. Identification of *M. tuberculosis* complex was done using rapid test SD BIOLINE TB Ag MPT64 Rapid, an immunochromatographic test that identifies *M. tuberculosis* complex via monoclonal anti-MPT64 mouse antibody. MPT64 is a specific protein of the *M. tuberculosis* complex*.*

### Patients information

Patient medical records were reviewed to obtain demographic data, HIV serology results, and a history of past or previous treatment of tuberculosis.

### Genotypic susceptibility testing

All isolates were freshly subcultured on liquid medium incubated in MGIT 960, before being tested. The Genotype MTBDRplus and MTBDRsl (Hain Lifescience, GmbH, Germany) were performed according to the manufacturer’s instructions. These are Line Probe Assays (LPAs) that are based on DNA strip technology. Genotype MTBDR*sl* was performed by an experienced operator, who received standardized training on the method and it has been done, routinely, in the past 4 years in our Lab. The test itself has internal quality control and the final results were double checked by two blinded experienced operators.

#### Genotype® MTBDRplus

MTBDRplus is a rapid molecular test that detects *M. tuberculosis* complex and mutations in: *rpoB* gene, this encodes RNA polymerase β-subunit, mutation in this gene causes resistance to rifampicin*; katG* gene (encodes catalase peroxidase enzyme) and *inhA* gene (encodes enoyl-acyl carrier protein redutase) mutation in these two genes causes resistance to isoniazid. We screened for mutations D516V, H526Y, H526D, and S531L in the *rpoB* gene, mutations S315T1, S315T2 in the *katG* gene, and mutations C15T, A16G, T8C and T8A in the *inhA* gene regulatory region. Any sample that had these mutations or absence of wild-type was categorized as resistant to the evaluated drug [15].

#### Genotype® MTBDRsl

The MTBDRsl assay identifies *M. tuberculosis* complex and important mutations that confer resistance to the second line Injectable anti-TB drugs (SLIDs), fluoroquinolones (FQ) and etambutol (EMB). To detect resistance for the SLIDs (kanamycin, amikacin and or capreomycin), the *rrs* gene was screened. For FQs (levofloxacin and ofloxacin), the *gyrA* gene was screened and for EMB the *embB* gene was screened. The following mutations A90V, S91P, D94A, D94N/Y, D94G, and D94H in the *gyrA* gene are associated with resistance to FQ. In the *rrs* gene, A1401G and G1484T mutations cause resistance to the SLIDs, while M306I and M306V mutations in the *embB* gene confer resistance to EMB. The strip contained conjugated control (CC) and amplification control band (AC). The MTBDRsl strip is also composed by six wild-type (WT) bands: three *gyrA* WT, two *rrs* WT and one *embB* WT. For interpretation of the strip, the presence of any mutation described above or absence of wild-type band was considered as indication of resistance to the evaluated drug [16, 17].

### Statistical analysis

Statistical test were performed using STATA 13.1 software (StataCorp LP College Station, Texas 77845 USA). Chi-square and Fisher exact test was used to determine differences between susceptible and resistant group. The results was considered significant for those having *p* < 0.05.

## Results

A total of 155 TB patients confirmed by culture and Genotype MTBDRplus were included. There were 77 (50 %) males, 62 (40 %) females and 16 (10 %) for whom gender was unknown. Pulmonary disease was diagnosed in 153 (98.7 %) patients. There were two cases of extra-pulmonary TB. TB/HIV coinfection was present in 61.1 % (44/72) of the 72/155 (46.5 %) patients who had HIV serology performed. *MTBDRplus results*: From the 155 isolates tested, 115 (74.2 %) where susceptible to isoniazid (H) and rifampicin (R) while any resistance to H or R was detected in 40 (25.8 %) isolates. The prevalence of MDR-TB meaning resistance to both R and H was 16.1 % (25/155), H-monoresistance (H-monoR) detected in 13 (8.4 %) isolates while R-monoresistance (R-monoR) occurred in 2 (1.3 %). Regarding to the previous use of anti-TB drugs, 4 (16 %) patients with MDR-TB had no previous treatment of TB, while 20 (80 %) patients reported previous TB treatment for more than 30 days. There was 1 (4 %) patient with unknown information about previous use of anti-TB drugs.

Among the population with any resistance to the evaluated drugs (40 isolates), the frequency of MDR-TB was 62.5 %, H-monoR 32.5 % and R-monoR 5 %, the frequency of primary DR-TB was 22.2 % (8) while in 77.8 % (28) the drug resistance was acquired (Table 1).

**Table 1:** Summary of *M. tuberculosis* susceptibility profile test based on Genotype MTBDRplus compared to gender, HIV co-infection and previous use of first line drugs against tuberculosis

	Gender				HIV				Previous treatment of TB				Total
*M. tb* status	M	F	Unk	*p* value	Pos	Neg	Unk	*p* value	Yes	No	Unk	*p* value	
Susceptible	55 71.4 %	49 79.0 %	11 (68.7)	0,33	29 66.0 %	20 71.4 %	66 79.6 %	0,80	73 72.2 %	33 80.5 %	9 69.2 %	0,02	115 74.2 %
MDR-TB	12 15.6 %	10 16.2 %	3 (18.8)		11 25.0 %	5 17.9 %	9 (10.8)		20 19.8 %	4 9.8 %	1 7.7 %		25 16.1 %
R-mono resistant	2 2.6 %	0	0 (0.0)		1 2.2 %	0	1 1.2 %		1 1.0 %	1 2.4 %	0		2 1.3 %
H-mono resistant	8 10.4 %	3 4.8 %	2 (12.5)		3 6.8 %	3 10.7 %	7 8.4 %		7 7.0 %	3 7.3 %	3 23.1 %		13 8.4 %
Total	77	62	16		44	28	83		101	41	13		155 100 %

*M. tb Mycobacterium tuberculosis*, *M* male, *F* female, *Unk* unknown, *Pos* positive, *Neg* negative, *MDR-TB* isolates resistant to rifampicin and isoniazid, *R-mono* resistant only to rifampicin, *H-mono* resistant only to isoniazid

*Chi-square* and *Fisher* comparing susceptible and non-susceptible groups. Significance *p* < 0.05

### Genotypic profile

Of the 27 isolates resistant to R with mutations detected in the *rpoB* gene, 37 % (10 isolates) presented S531L mutations; this was the most frequent profile (Table 2).

**Table 2:** Description and frequency of Genotype MTBDRplus detected mutations in *rpoB*, *katG* and *inhA* genes

*katG* Mutations	*inhA* Mutations	*rpoB* Mutations	Frequency	%
S315T1	-	-	9	22.5
S315T1	-	S531L	6	15.0
S315T1	-	H526Y	4	10.0
S315T1	-	S531L	2	5.0
S315T1	-	*WT	3	7.5
S315T1	-	*WT	2	5.0
S315T1	-	D516V	1	2.5
S315T1	-	*WT	1	2.5
S315T1	-	*WT	1	2.5
*WT	-	*WT	1	2.5
*WT	-	-	1	2.5
-		S531L	1	2.5
-	C15T	H526D	1	2.5
S315T1	C15T	*WT	1	2.5
S315T1	*WT	S531L	1	2.5
S315T1	C15T	-	1	2.5
-	C15T	-	1	2.5
-	C15T	*WT	1	2.5
-	-	H526Y	2	5.0
				100 %

*WT absence of one or more wild-type

-no mutation detected

### Genotype MTBDRsl

There were no discrepancies between the operators in the interpretation of the final result with the strip tests. A total of 125 isolates tested with MTBDRplus including the 25 isolates with resistance to R or H, were screened for resistance to the SLIDs, FQ and EMB using MTBDRsl test in other to determine the frequency of XDR-TB in this studied region of Mozambique. Of these, 109 (88 %) were susceptible while 16 (12 %) had any resistance detected. Within the resistant isolates, 37.5 % (6/16) were monoresistant to FQ, 56.25 % (9/16) monoresistant to EMB and 6.25 % (1/16) resistant to both FQ and EMB. There were no resistant detected to SLIDs as a consequence of this, XDR-TB did not occur in this sample. All isolates detected with resistance to EMB had previous use of anti-Tb drugs.

### Genotypic profile

The most frequent mutation detected in the isolates with resistance to FQ was A90V, this occurred in 100 % (7/7) while in those resistant to EMB loss of *embB* WT1 was more prevalent (90 %) (Table 3).

**Table 3:** Frequency of mutations detected by Genotype MTBDRsl in *embB* and *gryA* genes

*embB* Mutations	gryA Mutations	Frequency	%
*		6	37.5
*	-	1	6.25
M306I, M306V	-	1	6.25
M306I, M306V	-	1	6.25
M306I	A90V	1	6.25
-	A90V	4	25.0
-	A90V, D94G	1	6.25
-	A90V, D94N/Y, D94G	1	6.25
10	7	16	100 %

*absence of one or more wild-type

-no mutation detected

## Discussion

In Mozambique, surveillance for tuberculosis is poor [2, 18] and the true magnitude of drug resistance is unknown [2, 19]. Operations researches are needed to provide estimates of drug resistance and HIV coinfection burdens to improve treatment and prevention.

Our study is the first from Mozambique that provides an estimate the frequency DR-TB, coinfection DR-TB/HIV and the magnitude of resistance to SLIDs. Compared to national data, [7] our finding shows high frequency of resistance to the first line anti-TB drugs (R or H) (25.8 %) and high frequency of MDR-TB (16.1 %). Based on the history of previous therapy in the majority of our patients, drug resistant TB was mainly acquired as shown previously [6, 20]. However, the frequency of primary drug resistant TB (22.2 %) at Beira Hospital, in Central Mozambique may be an indication of failure to prevent transmission during management of DR-TB cases.

Because of the high prevalence of HIV in Mozambique, HIV infection was common among patients with DR-TB infection (62.5 %) although there is no association between HIV and drug resistant TB [21–23]. However, it was shown by the epidemics of DR-TB in Kwazulu Natal, South Africa (SA) that there is high case fatality among DR-TB/HIV co-infected patients [24, 25] so rapid diagnosis of drug resistance and guided treatment is important both for improved outcomes and to avoid progression to XDR-TB or “TDR-TB”. Genotypic molecular tests such as LPA (MTBDRplus, MTBDRsl), which are endorsed by WHO for surveillance of drug resistance have been revolutionary in shortening the time between DR-TB diagnostic and initiation of treatment [23]. In addition, these tests are improving the understanding of drug resistant TB worldwide and may also improve local protocols for treatment of DR-TB in settings with lack of specialized physicians for DR-TB management such as Mozambique.

It is expected that almost 95 % of strains resistant to R are also resistant to H, this supports the recommendation of WHO that all R resistant cases in regions with high prevalence of TB should receive treatment for MDR-TB [2], our findings support this recommendation because 93 % of R resistant isolates in our study were concomitantly resistant to H.

The first generation of Genotype MTBDRsl has low sensitivity especially for detection of resistance to SLIDs [10–13] but this test may be used for purpose of infection control and rule-in XDR-TB in settings such us Mozambique, where LAP technology is available and conventional DST capacity is limited. However, the test should not replace phenotypic DST as the gold standard for diagnosis of XDR-TB and clinicians must not rule out the investigation of XDR-TB when resistance is not detected on MTBDRsl [13]. This test has already a new version (MTBDRsl version 2.0) which shows better performance characteristics in detection of XDR-TB with high sensitivity and specificity, especially for kanamycin detection. This new test may shorten the time between diagnostic of DR-TB and initiation of treatment as well as estimates the prevalence of SLD resistance accurately [26].

In Mozambique there is a lack of information about resistance to the second line drugs. Samo Gudo et al., in their study using phenotypic DST within 41 strains obtained from national drug resistance surveillance, found no XDR-TB case in 2008 [6]. Pires et al., with 280 MDR-TB isolates using phenotypic DST, found only 2 (0.71 %) XDR-TB cases [20]. Using MTBDRsl we found low frequency of resistance to the SLD (12 %), almost 37.5 % (6/16) resistant to FQ, 56.3 % (9/16) to EMB and 6.3 % (1/16) resistant to both FQ and EMB, no resistance detected for SLIDs, and no XDR-TB case confirmed in this study. Based on these limited data, we suspect that XDR-TB is still infrequent in Beira Mozambique.

The major limitations of this study were the incomplete medical records, lack of information about phenotypic susceptibility tests and Genotype MTBDRsl low sensitivity to detect resistance to SLIDs.

This study is also part of an initiative to evaluate the cost/effectiveness of LPA test introduction in the drug resistant tuberculosis routine investigation at Beira, central Mozambique.

## Conclusions

Despite a high frequency of MDR, XDR-TB was not found in our patients. This study emphasizes the rational use of molecular tests for improving local protocols for treatment and prevention of drug resistant TB. Genotypic assays improve local information about SLD resistance in settings in which phenotypic testing is unavailable.

## Abbreviations

AC, amplification control; CC, conjugated control; DR-TB, drug resistant tuberculosis; DST, drug susceptibility test; EMB, etambutol; FQ, fluoroquinolones; H, isoniazid; HIV, human immunodeficiency virus; LPA, line probe assay; MDR, multidrug resistant; NPTBC, national program of TB control; R, rifampicin; SLD, second line drugs; SLIDs, second line Injectable anti-TB drugs; TB, tuberculosis; WHO, world health organization; WT, wild-type; XDR-TB, extensively drug resistant tuberculosis.
